# Fiscal transfers based on inputs or outcomes? Lessons from the Twelfth and Thirteenth Finance Commission in India

**DOI:** 10.1002/hpm.2444

**Published:** 2017-08-30

**Authors:** Victoria Y. Fan, Smriti Iyer, Avani Kapur, Rifaiyat Mahbub, Anit Mukherjee

**Affiliations:** ^1^ Center for Global Development Washington District of Columbia USA; ^2^ Office of Public Health Studies, Myron B. Thompson School of Social Work University of Hawai'i at Mānoa Honolulu Hawaii USA; ^3^ François‐Xavier Bagnoud Center for Health and Human Rights Harvard T.H. Chan School of Public Health Boston Massachusetts USA; ^4^ Accountability Initiative Center for Policy Research, Dharma Marg Chanakyapuri New Delhi India; ^5^ Tata Community Initiatives Trust Kala Ghoda Mumbai India; ^6^ J.F.K. School of Government Harvard University Cambridge Massachusetts USA

**Keywords:** fiscal federalism, India, intergovernmental fiscal transfers, public finance, results‐based financing

## Abstract

**Background:**

There is limited empirical evidence about the efficacy of fiscal transfers for a specific purpose, including for health which represents an important source of funds for the delivery of public services especially in large populous countries such as India.

**Objective:**

To examine two distinct methodologies for allocating specific‐purpose centre‐to‐state transfers, one using an input‐based formula focused on equity and the other using an outcome‐based formula focused on performance.

**Materials and Methods:**

We examine the Twelfth Finance Commission (12FC)'s use of Equalization Grants for Health (EGH) as an input‐based formula and the Thirteenth Finance Commission (13FC)'s use of Incentive Grants for Health (IGH) as an outcome‐based formula. We simulate and replicate the allocation of these two transfer methodologies and examine the consequences of these fiscal transfer mechanisms.

**Results:**

The EGH placed conditions for releasing funds, but states varied in their ability to meet those conditions, and hence their allocations varied, eg, Madhya Pradesh received 100% and Odisha 67% of its expected allocation. Due to the design of the IGH formula, IGH allocations were unequally distributed and highly concentrated in 4 states (Manipur, Sikkim, Tamil Nadu, Nagaland), which received over half the national IGH allocation.

**Discussion:**

The EGH had limited impact in achieving equalization, whereas the IGH rewards were concentrated in states which were already doing better. Greater transparency and accountability of centre‐to‐state allocations and specifically their methodologies are needed to ensure that allocation objectives are aligned to performance.

## 1 INTRODUCTION

In large, federal, populous countries such as the United States and India, fiscal transfers from central to state governments are an important source of funds for the delivery of public services.^1^, ^2^ When designed well, such intergovernmental fiscal transfers can increase the accountability and the effectiveness of public service delivery, which in turn can lead to improved outcomes in areas such as in health.^3^, ^4^ Although there is extensive theoretical knowledge about different fiscal transfer mechanisms, there is limited empirical evidence about the effectiveness and efficiency of these fiscal mechanisms especially with regards to improving health outcomes in low‐ and middle‐income countries.^5^

Two theoretical approaches have been applied to understanding these intergovernmental fiscal transfers. Traditional fiscal federalist theory assumes a perspective of the social planner, with the planner being the central government, and provides a normative framework for assigning responsibilities to different levels of entities.^6^, ^7^ Given the revenues raised through taxation, a given central government entity may face the challenge of distributing these funds across states and other subnational entities. Fiscal transfers can theoretically address two types of imbalances, vertical and horizontal. Vertical imbalances arise from the different ability of states compared to the central government in raising funds and carrying out their functional responsibilities, eg, public service delivery in health and education. Horizontal imbalances arise from the disparities across constituent units in the federation, ie, the states themselves, due to the different revenue‐raising abilities. Federal distribution of funds can thus either exacerbate or alleviate these disparities.

A second relevant framework is principal‐agent theory.^8^ In the case of intergovernmental fiscal transfers, a given central government entity may be seen as the principal and the state government as the agent. The principal (central government) has certain predefined goals, which are delegated to the agent (state government) to execute, and the agent is paid for completing certain activities and reaching these defined goals. Here, the systematic transfer of funds is often based on the receipt or verification of the *ex post* performance of certain indicators including submission of utilization certificates which may need to be submitted before receiving additional funds. Sometimes, however, the principal is unable to verify the outputs of its payments to the agent, or the outputs are not well defined and can be manipulated, and, consequently, the agent is able to shirk—by accepting funds without actually carrying out the mutually agreed upon activities and instead using the funds to their preferences.

Through the lens of a social planner, it can be argued that central governments have focused on the design and implementation of *ex ante* formula to allocate funds to its constitutive state treasuries and thereby address these two imbalances as an immediate goal of planning. Funds allocated based on *ex ante* formula can be channelled as general purpose transfers or unconditional block grants, thereby giving states the autonomy to allocate their budgets and determine their activities and expenditures, or alternatively transfers which focus on addressing vertical and horizontal imbalances. In contrast, principal‐agent theory lends itself to the use of conditional block grants or specific purpose transfers, with a focus on *ex post* conditionalities placed on activities completed or results achieved using the allocated funds. Both theories nevertheless lend to the use of formula or systematic methodology but vary in the timing of their payments and the type of data and information on which payments are determined.

This study adds to both of these literatures by using the case of specific‐purpose transfers by the Twelfth and Thirteenth Finance Commissions in India, one transfer using an input‐based formula and the other transfer using an outcome‐based formula. Finance Commissions are constitutional bodies set up every five years to provide recommendations on the division of tax revenue and grants between the central and state governments (see next section for further background on the Indian intergovernmental fiscal transfer system). For the purpose of this paper, we define inputs as the financial, human, and physical resources required to carry out an activity, whereas outputs and outcomes are measures and goals of an activity. To examine the input‐based formulaic approach, we study the case of the Twelfth Finance Commission (12FC)'s use of Equalization Grants for Health (EGH), for which the formula examined the needs and differences across states in their revenue capability (ie, their inputs). To examine the outcome‐based formulaic approach, we analyse the case of the Thirteenth Finance Commission (13FC)'s use of Incentive Grants for Health (IGH), for which the formula sought to incentivize states towards better performance for health outcomes (ie, their outcomes).

The goal of this study is to examine the extent to which both of these specific‐purpose transfers can be replicated using their predefined methodology and to investigate the advantages and disadvantages of these methodologies which, to our knowledge, has not been analysed or discussed hitherto in the literature. This paper is organized as follows. First, we describe the background to these specific‐purpose transfers of the 12FC and 13FC including international comparisons in the UK and US for additional contextual background. Next, we examine the methodologies and describe the data used. Our main results are a simulation and replication of these methods, with a focus on the distribution of funds across states and their equity and potential health implications. We conclude by offering specific policy recommendations to improve these types of mechanisms and policy alternatives.

## 2 POLICY BACKGROUND

### 2.1 Indian intergovernmental fiscal transfer system

Theoretical approaches are useful for providing intuition for prediction, but theories nevertheless should be contextualized to real‐world circumstances. As such, we briefly describe the complex intergovernmental fiscal transfer system in India and the political economy of these transfers to help elucidate the weaknesses of theory. Fiscal transfers from the centre to state in India represent a complex relationship between national and subnational governments and “are governed by a complicated mix of constitutional assignments, institutional precedents, discretion and negotiation.”^9^

The seventh schedule of the Indian Constitution assigns the specific powers and functions of the centre and the states. This list includes the Union list of the exclusive powers of the centre, the state list of exclusive powers of the states, and the concurrent list of those falling under the joint jurisdiction of both the centre and the states. Health in India is a state subject per the Constitution. However, amendments have given the Central government joint responsibility in areas such as population control, prevention of spread of infectious diseases, and regulation of food and drugs. This overlap of responsibility in financing of health between the central and state governments requires an effective and efficient system of fiscal transfers.

There are broadly three main channels of fiscal transfer systems. First, the Finance Commission, a constitutionally mandated body appointed by the President of India every 5 years, is responsible for the distribution of taxes between the centre and states. In addition to tax devolution, the Finance Commission is also required to recommend specific grants to the states in need of assistance.

The second main channel of fiscal transfers was through the now‐defunct Planning Commission—a central government body set up through an act of Parliament. Similar to the role of a social planner, the Planning Commission had allocative decision‐making power and responsible for dispensing assistance to states to finance development activities. Planning Commission transfers came through various channels: formula‐based normal central assistance, additional central assistance (mostly project financing in infrastructure), and special central assistance (on the basis of state‐specific priorities). States which were particularly disadvantaged were categorized as special category (as opposed to general category) and received additional funding. Past research found that allocations to states by Planning Commission were politically driven over 1987‐1995: states which elected leaders of the same party as the central government tended to receive more Planning Commission funds, whereas allocations by Finance Commission were not susceptible, if not countercyclical, to changes in political power.^10^

Finally, the third main channel is through various central ministries which give discretionary grants to their state counterparts, either wholly funded by the Centre (central sector programs) or requiring a matching state contribution known as Centrally Sponsored Schemes (CSS). An example of a CSS for health is the National Health Mission (NHM).

### 2.2 Indian health system in an international context

India's health system follows the model of the UK National Health Service (NHS) because a large part of the public health infrastructure was created during colonial time and expanded after independence in 1947. India's publicly delivered health services have suffered from underinvestment in personnel, infrastructure, and other inputs, leading to huge variations in service quality across states.^3^ The UK health system's use of target setting may provide a comparison for India's intergovernmental fiscal transfer system and in particular the 13FC's IGH. From 2001, NHS started publishing annual performance ratings for NHS trusts on a 3‐point scale. Trusts were evaluated on 3 key indicators—percentage of patients seen in emergency and accident wards within 4 hours; at least 75% of life threatening calls to ambulance services that were attended within 8 minutes; and reduction in the maximum wait time in the first elective hospital admission from the baseline of 18 months in 2001. These targets appeared to have been successful in improving health services; each of these indicators showed a positive trend between over 2001‐2005.^11^ The effect on services excluded from the ratings, however, is unclear, as there have been allegations of data manipulation and “gaming” to meet the prescribed targets.^12^ The move from structure and process indicators in the mid‐2000s towards outcome indicators at the end of the decade was in part due to the recognition that the latter are more effective at capturing improvements in the quality of services, a key goal of any health sector reform.

In contrast, financing for the US health system, specifically the Medicaid program, health insurance for low‐income households, provides a comparison for India's input‐based fiscal transfers such as the Twelfth Finance Commission's EGH and the Centrally Sponsored Scheme called the NHM. US Medicaid is financed using a formula called the Federal Medical Assistance Percentage (FMAP), for which the federal government pays a fixed share of all eligible Medicaid spending and is also called the “match rate”. The formula depends on the state's income level, which is a proxy for the state's ability to pay and raise revenues as well as the state's level of eligibility for the program, and is determined annually by comparing state's average per capita income level to the national average income level. According to this formula, the federal government shares range from 50% for higher‐income states to about 74% for lower‐income states, with the remainder covered by either state or local government.^13^, ^14^ Local government can include Medicaid providers (eg, a county hospital or school district) which either transfer funds to the state Medicaid agency through an intergovernmental transfer, or certify expenditures incurred to provide Medicaid services or administration, known as a certified public expenditure. The latter practice is analogous to the NHM for which states are expected to contribute 40% of total resources with centre providing 60% as of financial year 2015‐16. NHM payments, however, are not linked to insurance payments, similar to Medicaid certified public expenditures. While both EGH and IGH use a formula, neither EGH nor the IGH require matched state contributions as in Medicaid.

### 2.3 Twelfth Finance Commission's Equalization Grants for Health

Low and unequal public spending on social sectors including the health sector has long been identified by Government of India as a challenge.^15^ Indeed, CSS in India (such as NHM) were established precisely to increase spending by both state and central governments. The EGH established by the 12FC were also intended to address this problem of low public spending. See Annex 1 for per capita total public health expenditure prior to 12FC.

For the period 2005‐10, the 12FC was guided by “[a]n equalization principle in determining service‐specific grants can play an important role in a situation where, while the average expenditure on health and education may grow covering all states, for some states where service provisions are below average, expenditure on these heads needs to grow faster than the average, if they are to catch up.”^16^ Thus, the 12FC recommended two equalization grants to be distributed among states whose per‐capita expenditure was below the national average—one grant for the education sector amounting to Rs 10,171.65 crore (1 crore = 10 million) distributed to eight states and the other grant for the health sector amounting to Rs 5,887.08 crores for seven states.

Full capacity equalization would be constrained by a number of factors, including the sheer quantum of grants that would be required to achieve full equalization as well as presence of multiple channels of fiscal transfer, large and growing differences in state capacities, and problems regarding availability of accurate, real‐time data. Given these constraints, the 12FC focused on providing a partial correction in per‐capita expenditures for health and education. This was done in two ways. First, it allowed for expenditure restructuring in favour of each sector by providing higher growth rate for non‐salary component in projecting expenditure. Second, they provided separate grants‐in‐aid for certain states for health and education. Each of these is described in detail in Section 3 on methodology.

### 2.4 Thirteenth Finance Commission's Incentive Grants for Health

In contrast, the IGH established by the 13FC were intended to spur improvements in health outcomes and specifically further decrease infant mortality. Although formula‐based transfers have long been used by both the Finance and Planning Commission, much less is known about the use of formula in which the outputs or outcomes, rather than inputs, are used.

The 13FC guided by the principles of the “need to improve the quality of public expenditure to obtain better outputs and outcomes” recommended six different grants with incentives to improve outcomes as a supplement to its tax devolution and block grants, which totalled Rs 14,446 crore over the 5‐year period (2010‐15). Of these 6 grants for improving outcomes, one grant was allocated Rs 5,000 crore (35% of the total) for health specifically. This IGH used outcome‐based transfers with the goal of reducing infant mortality rates (IMR) in states. This novel use of outcome‐based transfers marked a departure from the 12FC which used an *ex ante* formula based on financial management and expenditure scores of states.

## 3 METHODOLOGY

### 3.1 12FC EGH

#### 3.1.1 Methodology

- *Expenditure Restructuring for Projecting Health Expenditures*

Projections for non‐plan health expenditures were made for the period 2005‐10. The first step was thus estimating base year figures for 2004‐05. In order to calculate this, the trend growth rate for 1993‐2003 was applied to the expenditure figures for 2002‐03 to arrive at the corresponding figures for 2004‐05. This was then compared with the budget estimates available for 2004‐05, and the lower of the two figures was taken as the base year estimates. Having determined the base year figures, the next step entailed determining the growth rate for the forecast period. It was found that, for the health EGH, on average 75% of expenditures consisted of salaries. In order to ensure that salaries are held constant in real terms, a 5% growth rate was assumed for the salary component of health expenditures. For the non‐salary component, a growth rate of 30% was assumed. Combining the growth rates of salary and non‐salary components with their respective weights as mentioned previously, a composite growth rate of 11.5% for health was obtained. These growth rates were then applied to states in order to obtain projected health expenditure for the period 2005‐10.

- *Grants‐in‐Aid for Specific States*

The next step was determining the eligibility of states who would receive the EGH and the quantum of grant to be provided. In devising the specific purpose grant for health, a two‐step normative approach was adopted. First, given that states accord different priority to the health as expressed in their total public health expenditure per capita, care was taken to not underwrite the deficiency in priority accorded to the sector by the state government by “correcting” for this low expenditure preference—see Table 1.

**Table 1:** Expenditure on medical and public health and family welfare as percentage of aggregate disbursements by state, 2001‐02

General category	Andhra Pradesh	4.4
	Bihar	4.9
	Chhattisgarh	4.3
	Goa	3.8
	Gujarat	2.8
	Haryana	3.0
	Jharkhand	4.9
	Karnataka	4.9
	Kerala	5.8
	Madhya Pradesh	4.1
	Maharashtra	4.3
	Odisha	3.7
	Punjab	3.9
	Rajasthan	5.2
	Tamil Nadu	4.9
	Uttar Pradesh	3.6
	West Bengal	5.0
Special category	Arunachal Pradesh	4.9
	Assam	4.2
	Himachal Pradesh	4.9
	Jammu and Kashmir	5.5
	Manipur	3.4
	Meghalaya	6.6
	Mizoram	5.4
	Nagaland	4.1
	Sikkim	2.2
	Tripura	3.7
	Uttarakhand	4.4

For this purpose, total expenditure data (both non‐plan and plan) for each of the states were examined for 2002‐03. The ratio of spending on health as a proportion of total expenditure was worked out for each state according to their categories (“special category” and “general category states”). In explaining the methodology, 12FC observed that “while working out this ratio, expenditure relating to pensions, interest payments and other adjustment items... were excluded from non‐plan revenue expenditure for arriving at the ‘adjusted’ total revenue expenditure.”

Next, the deviation of the average preference from the corresponding group average was calculated. For states whose proportionate expenditure on health was lower than the group average, the states proportion was replaced by the corresponding average group ratio. Having corrected for low expenditure preference, the next step entailed identifying those states which, even after spending the required percentage, fell short of comparable average per‐capita expenditure in the sector. This corrected per‐capita revenue expenditure for each state for 2002‐03 was thus calculated. Here, too, group averages were simultaneously worked out for general and special category states. The 12FC observed that those states whose per‐capita levels of public health expenditure were found to be below their group average “were reckoned as needing financial assistance, because their lower expenditure could be on account of low fiscal capacity.”^16^

For the states that fell in this category, due to limited availability of resources, the grant amount was calculated for covering 30% of the distance by which a below‐average state was lagging behind its group average of per capita expenditure. Finally, having determined the amount of grant required in 2002‐03 for health (multiplying the per‐capita requirement with the population figures), a growth rate equal to the group's TGR for 1993‐2003 for non‐plan revenue expenditure (NPRE) on health was applied on this amount in order to estimate the quantum of grant in the base year. The 12FC put the minimum amount to be received by an eligible state to Rs 10 crores per year as grant.

#### 3.1.2 Data sources

The primary data used for this exercise was the data from Finance Accounts available from state governments. In replicating this exercise, we have also used finance accounts data from 1993‐2010. Population data in calculating per‐capita health expenditure were taken from Census 2001.

### 3.2 Methodology and data for 13FC IGH

The methodology for the IGH was developed by the Administrative Staff College of India (ASCI) Hyderabad, which was tasked by the Finance Commission to design a formula‐based approach that could improve outcomes. ASCI's final methodology recognized that states each had different starting levels of performance, and their methodology used the median of state IMR as a key parameter in its formula. The intention of the formula was to provide greater incentives to states below the median state IMR.

The multi‐component formula depends on two factors: (1) whether there was progress in the parameter (ie, in reducing the IMR) (regardless of population weight); and (2) whether the improvement (ie, the reduction in IMR) was better than the median state IMR. The intention of these components was to encourage movement towards reducing IMR in general and to incentivize improvement below the median *level* of IMR. States were divided into two categories based on whether the baseline 2009 value is above or below the median IMR in 2009, and different formula were applied to each category in order to calculate a point value (called “incentive coefficient”) assigned to each state for the assessed year. To obtain the state's share of the IGH in the assessed year, the state's incentive coefficient was divided by the sum of the incentive coefficients of all states in that year. The absolute level of allocation was obtained by multiplying the allocation of the IGH for that year by the state's share. See Box 1 and Annex 2 for detailed explanation.

**BOX 1 Formula for determining state‐wise allocations of IGH**

**Table:** 

**Step 1.** Calculate each state's Incentive Coefficient. The median value for IMR (denoted as “M”) is computed for baseline year 2009. States are then divided into two broad categories: states above and below the median IMR in 2009. The assessed year is denoted as “*t*” and refers to either year 2011 or 2012.
*Case 1*. If the state is above the median value in 2009 in the base year
Scenario 1. If the state's IMR in the assessed year t decreased from its 2009 value (ie, IMR has improved) but remains above the 2009 baseline median: $\mathrm{IC}=100*\left\{\frac{{\mathrm{IMR}}_{2009}-{\mathrm{IMR}}_{t}}{{\mathrm{IMR}}_{2009}-M}\right\}$ We found 10 such states in 2011 and 8 such states in 2012.
Scenario 2. If the state's IMR in assessed year t decreased from its 2009 value (ie, IMR has improved) and is now below the median: $\mathrm{IC}=100+100*\left\{\frac{M-{\mathrm{IMR}}_{t}}{M}\right\}$ We found 4 such states in 2011 and 6 such states in 2012.
*Case 2*. If the state is below the median value in 2009
Scenario 3. If the state's IMR in the assessed year t remains below the median IMR and drops below their own 2009 value (ie, shows improvement), the state's IC is as follows: $\mathrm{IC}=100+\left\{100*\frac{{\mathrm{IMR}}_{2009}-{\mathrm{IMR}}_{t}}{{\mathrm{IMR}}_{2009}}\right\}*100*\left\{\frac{M-{\mathrm{IMR}}_{t}}{M}\right\}$ We found that there were 11 states in this category in 2011 and 12 states in 2012.
Scenario 4. If the state's IMR in the assessed year t increased from its baseline 2009 IMR but remains below the median, the state's IC is 100. We found 3 such states in 2011 and 2 such states in 2012.
In addition, there were two additional scenarios in which no incentive was to be given, ie, the state's IMR in the assessed year was worse than the baseline, and its baseline value was already above the median, or if the state's IMR in the assessed year was below the baseline median but its IMR increased above the baseline median.
**Step 2.** Sum all the Incentive Coefficients for all states.
**Step 3.** Divide each state's Incentive Coefficient by the sum of all Incentive Coefficients for that assessed year to obtain the state's share.
**Step 4.** Multiply the total allocation of the IGH for the assessed year by the state's share.

The IGH gave states a lead time in the first 2 years (2010 to 2011) to make improvements, and allocated the Rs 5,000 crore over the remaining 3 years (2012 to 2015), with Rs 1,500 crore each allocated in the first two years and the remaining Rs 2,500 crore for the final year (2014‐15). The annual payment was made only after publication of state‐wise IMR from the preceding year in the Sample Registration System (SRS) (described next). Each state's eligibility was determined annually, based upon their improvement in the IMR relative to their 2009 baseline value.

The Finance Commission used data on IMR from the SRS's Annual Bulletin and Report, executed by the Office of the Registrar General and Census Commissioner. Beginning on a pilot basis from 1964‐65 and on a full‐scale regular basis since 1969‐70, the SRS is an independent source of nationally representative information on demographic outcomes including mortality and morbidity of Indian households. The 2011 sampling frame collected data longitudinally from 7,597 households. To replicate the IGH formula, we obtained publicly available reports with IMR data for the relevant calendar years (2009, 2011, and 2012)—see Table 2.

**Table 2:** Infant mortality rates by state and calendar year

State or union territory	Baseline: 2009	Year 1: 2011	Year 2: 2012
Andhra Pradesh	49	43	41
Arunachal Pradesh	32	32	33
Assam	61	55	55
Bihar	52	44	43
Chhattisgarh	54	48	47
Goa	11	11	10
Gujarat	48	41	38
Haryana	51	44	42
Himachal Pradesh	45	38	36
Jammu and Kashmir	45	41	39
Jharkhand	44	39	38
Karnataka	41	35	32
Kerala	12	12	12
Madhya Pradesh	67	59	56
Maharashtra	31	25	25
Manipur	16	11	10
Meghalaya	59	52	49
Mizoram	36	34	35
Nagaland	26	21	18
Odisha	65	57	53
Punjab	38	30	28
Rajasthan	59	52	49
Sikkim	34	26	24
Tamil Nadu	28	22	21
Tripura	31	29	28
Uttar Pradesh	63	57	53
Uttarakhand	41	36	34
West Bengal	33	32	32

*Note:* IMR data for the calendar year are obtained from the Annual SRS Report in the subsequent year.

## 4 RESULTS

### 4.1 12FC EGH results

A comparison of the projected and actual NPRE for the period 2005‐10 indicates interstate differences (Table 3). In 2005‐06 for instance, while actual NPRE in Maharashtra was significantly higher than the projected (difference of Rs 66 crores), in contrast, actual NPRE in Tamil Nadu were significantly lower than projected NPRE. The gap between actual expenditures and projected expenditures was highest in 2009‐10. Overall, while there was an absolute difference of Rs 844.25 crores between actual and projected NPRE in 2005‐06, the difference increased to Rs 2,993.13 crores in 2009‐10.

**Table 3:** Differences in actual expenditure and projected expenditure, 2005‐10

	State	2005‐06	2006‐07	2007‐08	2008‐09	2009‐10
Special category states	Arunachal Pradesh	6.14	16.10	8.29	39.69	86.68
	Himachal	0.09	2.65	16.00	230.46	327.04
	Jammu and Kashmir	−28.16	13.85	106.68	118.41	226.44
	Manipur	6.59	−8.48	−1.38	1.04	−6.79
	Meghalaya	1.24	−5.52	−4.99	−2.99	28.19
	Mizoram	−4.55	−8.75	−0.41	3.87	7.66
	Nagaland	19.86	29.49	32.71	27.74	35.87
	Sikkim	2.62	0.39	2.73	6.95	31.63
	Tripura	−6.41	−7.88	−16.12	−17.61	0.86
General category states	Andhra Pradesh	−3.51	31.07	271.54	235.65	118.39
	Chhattisgarh	12.64	−17.62	−35.66	−21.29	23.64
	Goa	2.27	3.00	4.42	40.10	62.13
	Gujarat	−5.01	−63.65	−99.08	−177.24	−183.14
	Haryana	0.60	−16.76	14.11	110.95	253.84
	Karnataka	−56.61	−102.89	19.84	−11.73	−80.62
	Kerala	−122.77	−76.33	−65.74	137.31	22.56
	Maharashtra	66.00	14.18	−18.24	64.67	411.75
	Punjab	−97.21	−174.79	−211.63	−263.85	−235.05
	Rajasthan	−53.92	−109.63	−139.84	167.44	272.73
	Tamil Nadu	−203.10	−133.90	−44.49	152.05	247.28
	West Bengal	−144.95	−224.63	−263.25	−294.42	330.84

Notes: Values are in crore rupees. Authors' calculations. Projections are from the 12FC report and actual expenditure from Finance Accounts. Refer to Annex 3, Table A3‐2 for details on EGH states.

The 12FC had projected a growth rate of 11.5% over 2005‐10. We calculated the growth rate based on actual NPRE to gauge whether the actual growth rates were close to the 11.5% estimate. The 12FC estimate of 11.5% growth rate was a conservative estimate for many states (see Annex 3, Table A3‐1). Moreover, there was significant variation both across states and between years in growth rates. In Arunachal Pradesh, while the growth rate between 2005‐06 and 2006‐07 was 29%, this dropped to a negative growth rate the following year. In the last 2 years of the 12FC period, the growth rate was over 50%. In Tamil Nadu in contrast, the growth rate was relatively stable year on year at around 20%.

Finally, the 12FC recommended an amount of Rs 5,887.08 crores spread across seven states of India. Annex 3, Table A3‐2 presents the year‐wise quanta of grants. Uttar Pradesh and Bihar received the maximum together, constituting 70% of the total EGH grants. Uttarakhand in contrast received the minimum of Rs 10 crores per year.

In short, we were unable to replicate the grants‐in‐aid to the specific seven states due to data constraints. The 12FC methodology note mentioned a number of adjustments undertaken in the NPRE. While some of these such as pensions, lotteries, calamity relief, and interest payments were easy to identify, it was difficult to cull out all the adjustments made to the NPRE. As a consequence, only a partial replication could be made, and the analysis has thus been omitted from this study.

Despite the difficulties of replication, the relative amount of allocation to states merits consideration. The EGH for the seven states had a number of conditionalities attached in terms of the NPRE that should be incurred, and consequently not all grants‐in‐aid were ultimately released (see Annex 3, Table A3‐3). For example, whereas Madhya Pradesh got its entire allocation, Odisha only received 67% of its allocation. When examining the 12FC EGH allocations as a proportion of actual total NPRE, there is considerable variation, suggesting that that the nature of the EGH formula will be limited in how much equalization can be achieved (see Annex 3, Table A3‐4). Moreover, projections can be either overestimates or underestimates of actual spending.

### 4.2 13FC IGH results

Using the IMR values presented in Table 2, we replicated the 13FC's formula for IGH and calculated each state's expected share of total allocations for the first 2 years of IGH's implementation—see Table 4. Table 4 shows that one state alone, Manipur, received more than a quarter and more than a fifth of the national allocation in the 2 years. Four states (Manipur, Sikkim, Tamil Nadu, and Nagaland) received over half of the national allocation, in each of the 2 years. The formula incentivizes states that are already below the median by offering a higher share of the total allocation. In 2012 and 2013, better‐performing states who were already below the median level, received approximately 57% of the national allocation of the total IGH and in 2013 they received 56% of the grant amount.

**Table 4:** State allocation of 13FC IGH as percentage of national allocation, 2012‐14

State or union territory	Change in IMR		State allocations of IGH, 2012‐13		State allocations of IGH, 2013‐14	
	2009‐11	2009‐12	% Share	Rs Crore	% Share	Rs Crore
Andhra Pradesh	6	8	1%	14	1%	12
Arunachal Pradesh	0	−1	1%	15	1%	11
Assam	6	6	0%	5	0%	4
Bihar	8	9	1%	13	1%	11
Chhattisgarh	6	7	1%	8	0%	7
Goa	0	1	1%	15	6%	89
Gujarat	7	10	1%	16	1%	12
Haryana	7	9	1%	13	1%	11
Himachal Pradesh	7	9	1%	17	1%	13
Jammu and Kashmir	4	6	1%	16	1%	12
Jharkhand	5	6	1%	16	1%	12
Karnataka	6	9	4%	55	5%	72
Kerala	0	0	1%	15	1%	11
Madhya Pradesh	8	11	0%	5	0%	5
Maharashtra	6	6	9%	137	7%	100
Manipur	5	6	25%	368	22%	332
Meghalaya	7	10	0%	6	0%	7
Mizoram	2	1	2%	32	1%	17
Nagaland	5	8	11%	164	14%	209
Odisha	8	12	0%	5	0%	6
Punjab	8	10	7%	110	7%	112
Rajasthan	7	10	0%	6	0%	7
Sikkim	8	10	10%	155	10%	154
Tamil Nadu	6	7	12%	173	10%	153
Tripura	2	3	3%	46	3%	48
Uttar Pradesh	6	10	0%	4	0%	5
Uttarakhand	5	7	3%	44	3%	49
West Bengal	1	1	2%	27	1%	20

*Note:* The change refers to the difference from the baseline year (2009) to the assessed year and hence is positive when IMR is reduced. Over the period of interest, IMR reduced or stated the same in all states, except for Arunachal Pradesh, where it increased. IMR 2011 data are used for 2012‐13 IGH, and IMR 2012 data are used for 2013‐14 IGH.

Whereas the formula was constructed purely based on performance of IMR relative to different standards, the allocation levels were not benchmarked according to either the level of health expenditure or the population. Indeed, given the capture of over half of the winnings of the total national allocation, ie, Rs 1,500 crores for four states in each of the first two years, the question arises regarding the size of these winnings relative to the total state health expenditure budget. Alternatively, the allocated funds can be benchmarked relative to the population, to give a sense of the leverage that the incentive might (or might not) have provided.

For the first benchmark, we calculated the state's IGH allocation as a percentage of the total state health expenditure (note that data obtained from the Reserve Bank of India excluded off‐budget NRHM releases to State Health Societies). Although data on actual state health expenditure were not available for 2012‐13 and 2013‐14, we use revised estimates for 2012‐13 and budgeted estimates for 2013‐14 as a proxy for actual expenditure. Table 5 shows that in most cases the allocations of the 13FC IGH constituted less than 2% of the state's total health spending. By contrast, in Manipur, Nagaland, and Sikkim, the 13FC IGH alone comprised 88%, 72%, and 60% of the state's health expenditure budget, respectively. As a check, we used an alternate comparator of state health expenditure budget plus NRHM releases and found similar results.

**Table 5:** 13FC IGH allocation as percentage of state's total health expenditure, 2012‐14

State or union territory	13FC IGH as percentage (%) of total health expenditure		13FC IGH per capita*(rupees per person)	
	2012‐13	2013‐14	2012‐13	2013‐14
Andhra Pradesh	0.2	0.2	0.17	0.14
Arunachal Pradesh	4.6	4.0	11.02	8.08
Assam	0.3	0.2	0.16	0.12
Bihar	0.4	0.3	0.12	0.10
Chhattisgarh	0.5	0.3	0.31	0.27
Goa	3.0	16.9	10.45	60.94
Gujarat	0.3	0.2	0.26	0.20
Haryana	0.7	0.6	0.50	0.45
Himachal Pradesh	1.6	1.1	2.46	1.88
Jammu and Kashmir	0.9	0.6	1.26	0.96
Jharkhand	1.1	0.9	0.50	0.37
Karnataka	1.3	1.3	0.89	1.18
Kerala	0.5	0.3	0.46	0.33
Madhya Pradesh	0.1	0.1	0.07	0.07
Maharashtra	2.0	1.4	1.22	0.89
Manipur	103.0	87.7	128.98	116.15
Meghalaya	1.6	1.5	2.18	2.28
Mizoram	14.4	9.9	29.33	15.18
Nagaland	53.2	71.6	82.66	105.86
Odisha	0.3	0.3	0.13	0.14
Punjab	4.0	3.9	3.95	4.02
Rajasthan	0.2	0.1	0.09	0.10
Sikkim	61.1	60.0	253.04	252.68
Tamil Nadu	3.1	2.5	2.40	2.11
Tripura	8.8	9.7	12.65	13.09
Uttar Pradesh	0.0	0.1	0.02	0.03
Uttarakhand	3.3	3.7	4.33	4.89
West Bengal	0.6	0.4	0.29	0.21

For a second benchmark, we divided the state's IGH allocation by the state's population in 2011‐12. Similar to the total state health expenditure variable, the distribution of per capita IGH allocations suggests that the vast majority of states had less than one rupee per person allocated for reducing infant mortality, whereas the three winning states of Manipur, Nagaland, and Sikkim each received 116, 106, and 253 rupees per person, respectively, in 2013‐14. This indicates the need to reassess the incentive conditionalities by incorporating population weights, as well as the underlying data used to calculate the quantum of transfers as per the formula.

## 5 DISCUSSION AND CONCLUDING REMARKS

In this study, we analysed the two cases of the 12FC and 13FC's use of two different specific‐purpose transfers for health. We found that EGH placed conditions for releasing funds, but states varied in their ability to meet those conditions, and hence their actual releases varied considerably, eg, Madhya Pradesh received 100% and Odisha 67% of its expected allocation.

We found that the 13FC IGH allocations revealed highly unequal distribution of ‘winnings’ and raises several questions about the efficacy and implications on equity of the formula. The formula's design gave a particularly large reward to those states which were already doing better, ie, states which were already below the median level for IMR. We found that the implementation of the formula generated a perverse scenario in which the vast majority of the grants went to four states alone. Further, we found that the absolute level of allocations relative to certain benchmarks were unreasonable, ie, compared to the state's health expenditure and the state's population. Whereas the four winning states received between 100 to 200 rupees per person under the IGH, most states received less than 1 rupee per person.

Several challenges and recommendations are shared for the two methodologies. First, the lack of a clear methodology as well as readily accessible data make it difficult to simulate and replicate the results for both methodologies, with the 12FC EGH being arguably more complex than the 13FC IGH. Furthermore, the 12FC EGH's rationale for choosing the eligible seven states was not transparent. By making eligibility criteria more transparent, states could be incentivized to increase their eligibility status. The ability to replicate these allocations using a transparent methodology and publicly available data can help to ensure greater integrity and robustness of the allocations and reduce the risk of poor distribution in consequences that were seen. One general recommendation is to encourage formula‐based centre‐to‐state allocations and to make their data and methodology replicable and available to states and the public at large. In general, greater transparency of the methodology of the Finance Commission reports as well as data collected and analysed are needed.

Despite these design challenges in both the 12FC and 13FC, formula‐based transfers in India are more important today than in the recent past. A third type of allocation is that which is not based on any formula and often are discretionary to political decisions. Grants determined by the Finance Commission have been predominantly based on formula, either for general purpose by the state treasury “Tax Devolution and Block Grants” or for specific purpose, eg, “Equalization Grants” or “Incentive Grants”, with a fraction of funds going for state‐specific grants (which are not based on formula).^17^

By no means should the results of the 13FC IGH replication be a rejection of *all* outcome‐based fiscal transfers. Rather, the particular design of this fiscal transfer is clearly problematic and with a severely perverse distribution. The complex formula also presented several highly implausible counterfactual scenarios, eg, providing no incentive to the better‐performing states if their IMR increased (highly unlikely, because they were already on a trajectory to reduce their IMR). The lack of population weight or weight by the number of babies born or babies who died was also problematic and contributed to this severely unequal distribution of payments captured by small better‐performing north‐eastern states.

There are many alternatives to structuring the formula of outcome‐based fiscal transfers. A simpler formula is more likely to convey clear information to states about what is required of them and what they might feasibly be rewarded with for their efforts.^18^ One such alternative would be to simply recognize that most states are already on a trajectory of reducing infant mortality, even without any additional incentive. The challenge is thus to further bend the curve of infant mortality reductions especially in those states that have the highest attainment gap compared to the better performing ones. Therefore, states should be rewarded for improving their IMR at a rate higher than their expected trajectory of IMR decrease. By plotting IMR over time, a state's IMR can be reasonably predicted. For each additional averted infant death beyond this expected trajectory, the state can be compensated for a fixed price. For example, states which expected an annual IMR reduction of 3 births per annum should be rewarded if they achieve a reduction greater than their expected decline, benchmarked by the expected number of infant deaths averted.^3^ How the budget envelope is defined (eg, on a per unit basis) and whether the states can collect savings (eg, if states can deliver cheaper programs while maintaining coverage) are critical design parameters. The US Medicare Shared Savings program, for example, incentivizes states in the form of an increased share of the savings and also reflects a global budget payment mechanism potentially linked to specific quality indicators.^19^

Despite the perverse distributional consequences of this formula, the 13FC should be lauded for their boldness and willingness to experiment with such an innovative fiscal transfer mechanism and should continue to experiment and learn.^20^ In addition, the IGH's use of an independent data source—namely, the SRS—is a substantial improvement over the use of self‐reported administrative data, which are subject to bias.

This study's results also raise questions with regards to the efficacy and efficiency of different transfer mechanisms in a context with multiple channels of delivery and usage. For instance, all the states which received the EGH also received additional funds under the National Rural Health Mission being part of the group that was lagging behind in health indicators compared to the rest of the country. In addition, there are general purpose transfers to states both by the Finance Commission and Planning Commission. These multiple sources of funds with their independent criteria and release mechanisms can at times lead to a fragmentation of transfers, which in turn can fragment state's attention.^21^

These specific‐purpose transfers do not tackle the challenge of fungibility. Past work has shown that central‐to‐state allocations results in state's lower contributions in that sector.^22^ Comparable issues of fungibility and lowering domestic spending in response to international aid have been observed in the context of international‐to‐national aid relationships.^23^

To get better outcomes, international experiences suggest that transfers need to consider at least three key dimensions. First, central government's allocation of national revenues to subnational governments should correspond to population size and needs, however defined. Second, transfers should generate incentives to improve the quality of the spending by subnational governments and their subsequent performance on outcomes. Third, independent systems to monitor, evaluate, and provide feedback data on subnational performance can generate greater accountability to the central government, parliaments, and legislatures, and ultimately greater accountability to the public. Improving performance of intergovernmental fiscal transfers may well require greater transparency and greater accountability.

Beyond the designs of outcome‐based fiscal transfers, future work should go further down below the level of state, ie, how to allocate transfers to districts and ultimately households. The Fourteenth Finance Commission, the most recent one, submitted its report in February 2015.^24^ In the context of this Finance Commission's recommendations which significantly increased the share of untied transfers to states, both the central government and states could consider the use of such specific‐purpose transfers to *districts*.

Data at the district level is hardly in short supply in India. The District Level Health Survey conducted by the International Institute for Population Sciences collects representative data every five years about reproductive and child health in all Indian districts. Moreover, the newer Annual Health Survey conducted at district level by the Office of the Registrar General and Census Commissioner represents an important source of independent, unbiased, and accurate information about mortality in lower‐performing districts. Together, these two sources of information could be utilized to develop district‐level outcome‐based fiscal transfers.

## FUNDING

Bill and Melinda Gates Foundation.

## ETHICS STATEMENT

Only publicly available budget data are used for this study. As no human subject data was used for this study, no approval by an institutional review board was necessary.

## 1

### ANNEX 1 PUBLIC HEALTH EXPENDITURE PER CAPITA, 2004‐2005

**Table:**

State	Per‐capita total public health expenditure, 2004‐05 (rupees)
Andhra Pradesh	191
Arunachal Pradesh	841
Assam	162
Bihar	93
Chhattisgarh	146
Goa	861
Gujarat	198
Haryana	203
Himachal Pradesh	630
Jammu and Kashmir	512
Jharkhand	155
Karnataka	233
Kerala	287
Madhya Pradesh	145
Maharashtra	204
Manipur	294
Meghalaya	430
Mizoram	867
Nagaland	639
Odisha	183
Punjab	247
Rajasthan	186
Sikkim	1082
Tamil Nadu	223
Tripura	328
Uttar Pradesh	128
Uttarakhand	280
West Bengal	173

### ANNEX 2 FORMULA FOR 13FC IGH

The detailed formula is described below and obtained from Finance Commission's Report, Annexure 12.10, with additional comment by authors.

1. The methodology employed for awarding points to states (and determining incentives) is based on the following premises: (1) initial conditions of all states should be taken due note of; (2) the improvement (or deterioration) in their performance over their level in the base year (initial condition) should be duly rewarded (or penalized); (3) states that are above the benchmark level should receive a minimum level of points *plus* additional points for improved performance, if any, during the period under consideration; and (4) the higher the level of performance in the base year over the benchmark, improvement over their base level (initial condition) would be that much harder and should therefore receive ‘elevated weightage’.
2. States would be awarded points based on their incremental performance over the base year in relation to (1) their initial condition and (2) the predetermined standard or benchmark. Initial condition is defined as the (output or outcome or any other indicator) performance level of a state in the base year. Incremental performance is the difference between the performance level in the year of reckoning (terminal year) and the performance level in the base year (initial year).
3. The points earned by states on this basis (which can be termed incentive coefficient) would be aggregated, and each state's points (incentive coefficient) would be calculated as a percentage of this aggregated total, which would be the state's incentive value or incentive percentage. States would then be eligible for incentive grants on the basis of this incentive percentage.
4. The rationale is as follows: states that have attained *relatively* higher levels of performance and are at the high end of the “performance spectrum” would have *comparatively* restricted scope for further percentage improvement over the base year level. The intention is that states that are already at a *relatively* higher level of performance and are to some extent disadvantaged by the restricted scope for incremental percentage improvement should not stand to lose. Hence, their percentage improvement in performance over the base (or initial) year should be suitably weighted to compensate them for this “inherent disadvantage”. It is, therefore, proposed to weight their performance by the distance of their output/outcome indicator from the median (benchmark) as a percentage of the median (benchmark).
5. For Infant Mortality Rate there is an inverse relation between the level of the indicator and performance of the state. Ie, a decrease in the indicator will lead to an incentive, while an increase will be penalized. The formula incorporates this requirement.

### ANNEX 3 ADDITIONAL 12FC ANALYSIS

**Table A3‐1:** Actual growth rates in expenditures, 2005‐10

Category	States	2005‐06 to 2006‐07	2006‐07 to 2007‐08	2007‐08 to 2008‐09	2008‐09 to 2009‐10
Special Category States	Arunachal Pradesh	29%	‐3%	57%	52%
	Himachal	13%	19%	115%	27%
	J&K	25%	33%	11%	27%
	Manipur	−13%	26%	15%	2%
	Meghalaya	0%	13%	15%	51%
	Mizoram	−1%	43%	22%	18%
	Nagaland	21%	11%	3%	16%
	Sikkim	3%	19%	22%	64%
	Tripura	10%	1%	12%	37%
General Category States	Andhra Pradesh	15%	30%	7%	3%
	Chhattisgarh	−5%	3%	21%	32%
	Goa	12%	13%	44%	23%
	Gujarat	4%	8%	3%	13%
	Haryana	5%	22%	37%	37%
	Karnataka	6%	28%	8%	5%
	Kerala	20%	14%	32%	2%
	Maharashtra	8%	10%	16%	27%
	Punjab	1%	9%	7%	19%
	Rajasthan	6%	10%	43%	17%
	Tamil Nadu	21%	21%	26%	16%
	West Bengal	6%	10%	11%	54%

**Table A3‐2:** Grants in aid for health

States	2005‐06	2006‐07	2007‐08	2008‐09	2009‐10	2005‐10 (Total)
Assam	153.58	171.24	190.93	212.89	237.38	966.02
Bihar	289.3	322.57	359.66	401.02	447.14	1819.69
Jharkhand	57.39	63.99	71.35	79.55	88.7	360.98
Madhya Pradesh	28.88	32.2	35.9	40.03	44.63	181.64
Odisha	31.22	34.81	38.81	43.28	48.25	196.37
Uttar Pradesh	367.63	409.9	457.04	509.6	568.21	2312.38
Uttaranchal	10	10	10	10	10	50
Total	938	1044.71	1163.69	1296.37	1444.31	5887.08

**Table A3‐3:** Proportion of EGH allocations released

State	Allocation	Releases	Proportion of allocations released
Assam	966.02	870.555	90%
Bihar	1819.69	1439.35	79%
Jharkhand	360.98	276.855	77%
Madhya Pradesh	181.64	181.64	100%
Odisha	196.37	131.2	67%
Uttar Pradesh	2312.38	1829.06	79%
Uttarakhand	50	40	80%
Total	5887.08	4768.66	81%

**Table A3‐4:** Grants received as a proportion of actual total NPRE

State	2005‐06	2006‐07	2007‐08	2008‐09	2009‐10
Assam	54.39%	36.50%	37.18%	32.09%	23.49%
Bihar	38.15%	37.96%	35.56%	38.58%	37.67%
Jharkhand	15.75%	22.17%	22.81%	19.57%	18.63%
Madhya Pradesh	4.13%	4.19%	3.88%	4.12%	3.77%
Orissa	7.21%	7.19%	7.37%	6.28%	5.73%
Uttar Pradesh	20.35%	20.79%	19.98%	19.23%	15.80%
Uttarakhand	5.72%	5.71%	5.02%	3.54%	2.87%
